# Adult Pott’s Puffy Tumor in the ED

**DOI:** 10.7759/cureus.117350

**Published:** 2026-10-02

**Authors:** Kim Rawlings, James Ross, Patricia Monteiro

**Affiliations:** 1 Internal Medicine, Great Western Hospitals NHS Foundation Trust, Swindon, GBR; 2 Interventional Radiology, Gloucestershire Hospitals NHS Foundation Trust, Gloucester, GBR; 3 Emergency Medicine, Great Western Hospitals NHS Foundation Trust, Swindon, GBR

**Keywords:** dental focal infection, frontal bone osteomyelitis, frontal sinus infection, pott's puffy tumor, sinonasal infection

## Abstract

Pott’s puffy tumor (PPT) is a rare but potentially serious complication of sinonasal infection characterized by frontal bone osteomyelitis with an associated subperiosteal abscess. Although traditionally associated with adolescents, it can occur in adults and may present in the absence of systemic features of infection. This can create a diagnostic challenge in the ED, where frontal swelling may initially appear to represent a more superficial or uncomplicated process. We present a case of a 50-year-old man who attended a district general ED with a prolonged history of sinonasal symptoms and a previous odontogenic infection, followed by the development of significant frontal swelling. Despite extensive local pathology, there were no systemic, neurological, or visual abnormalities. The diagnosis prompted early antimicrobial treatment and urgent referral for specialist surgical management, with an uncomplicated recovery. This case demonstrates the importance of recognizing PPT as a potential complication of sinonasal and dental infection in adults. Emergency clinicians should remain alert to the significance of frontal swelling, even in patients who appear systemically well. Early recognition, appropriate initial management, and timely specialist transfer are essential to prevent potentially serious intracranial complications.

## Introduction

Frontal swellings presenting to the ED have a broad differential diagnosis, ranging from benign dermatological conditions and localized soft tissue infection to pathology involving the underlying bone and intracranial structures. In the context of preceding sinonasal symptoms, the possibility of a complication, such as intracranial extension, arising from infection should be considered. Pott’s puffy tumor (PPT) is an uncommon but important cause of frontal swelling and can be defined as osteomyelitis of the frontal bone associated with a subperiosteal abscess. Due to its rarity, the condition may be unfamiliar to emergency clinicians and could be overlooked when patients lack systemic features of infection [1].

Although traditionally associated with adolescents because of increased vascularity of the developing frontal sinus, PPT is increasingly being reported in adults. A systematic review of adult cases identified a median age of 45 years, demonstrating that the condition should not be regarded exclusively as a disease of younger patients [2]. In addition to frontal sinusitis, the condition has also been associated with odontogenic infection, which may contribute to the development of PPT through contiguous spread [3].

We report a case of PPT in a 50-year-old man presenting to the ED, highlighting the diagnostic considerations associated with unusual frontal swelling in an otherwise systemically well adult and the importance of early recognition in the acute setting.

## Case presentation

A 50-year-old man presented to the ED with a several-month history of sinus congestion, offensive rhinorrhea, and cacosmia. Eight months before presentation, he had undergone extraction and washout of a right upper dental abscess. Over the preceding four weeks, he had developed progressively enlarging frontal swelling over his forehead, which was erythematous, fluctuant, and painful.

On examination, he was apyrexial and hemodynamically stable, with physical observations within normal limits. A large, 4 × 4 cm, fluctuant, erythematous swelling was present over the central forehead (Figure 1). Cranial examination was normal. The pupils were equal and reactive to light, with no focal neurological deficits or visual abnormalities detected.

**Figure 1:**
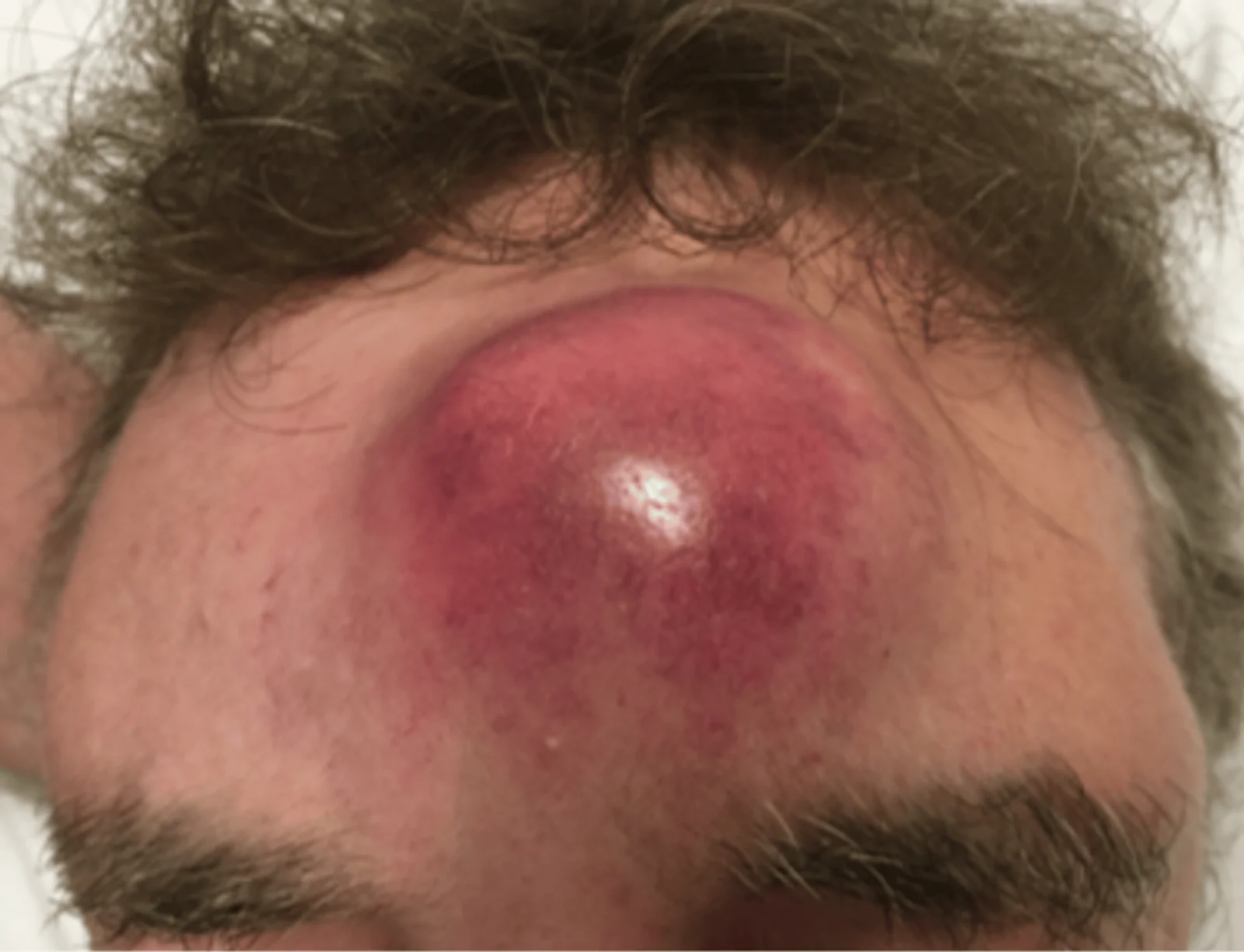
Clinical presentation in the ED, showing a large, fluctuant, erythematous swelling on the forehead.

Initial blood test results are shown in Table 1. Laboratory investigations demonstrated leukocytosis (16.9 × 10⁹/L), neutrophilia (13.47 × 10⁹/L), and a moderately elevated CRP (45 mg/L). Despite these abnormalities, the degree of inflammatory marker elevation was relatively modest compared with the extent of disease identified on imaging. Microbiological sampling was not obtained during the patient’s initial ED assessment.

**Table 1:** Initial blood test results.

Blood test	Result	Reference range
CRP	45 mg/L	<5 mg/L
Total WBC	16.9 × 10⁹/L	4.0-11.0 × 10⁹/L
Neutrophils	13.47 × 10⁹/L	2.0-7.5 × 10⁹/L
Platelets	213 × 10⁹/L	150-400 × 10⁹/L

Contrast-enhanced CT of the head and paranasal sinuses demonstrated a 3.5 × 2.5 cm anterior frontal collection consistent with PPT. There was expansion of the frontal sinus with low-attenuation material consistent with a mucocele, with defects in the sinus wall (Figure 2A, 2B). Posteriorly, there was a smaller defect within the left frontal sinus wall. There was also evidence of a mucocele in the right maxillary sinus bulging into the nasal cavity, with erosion of the medial wall of the maxillary sinus.

**Figure 2:**
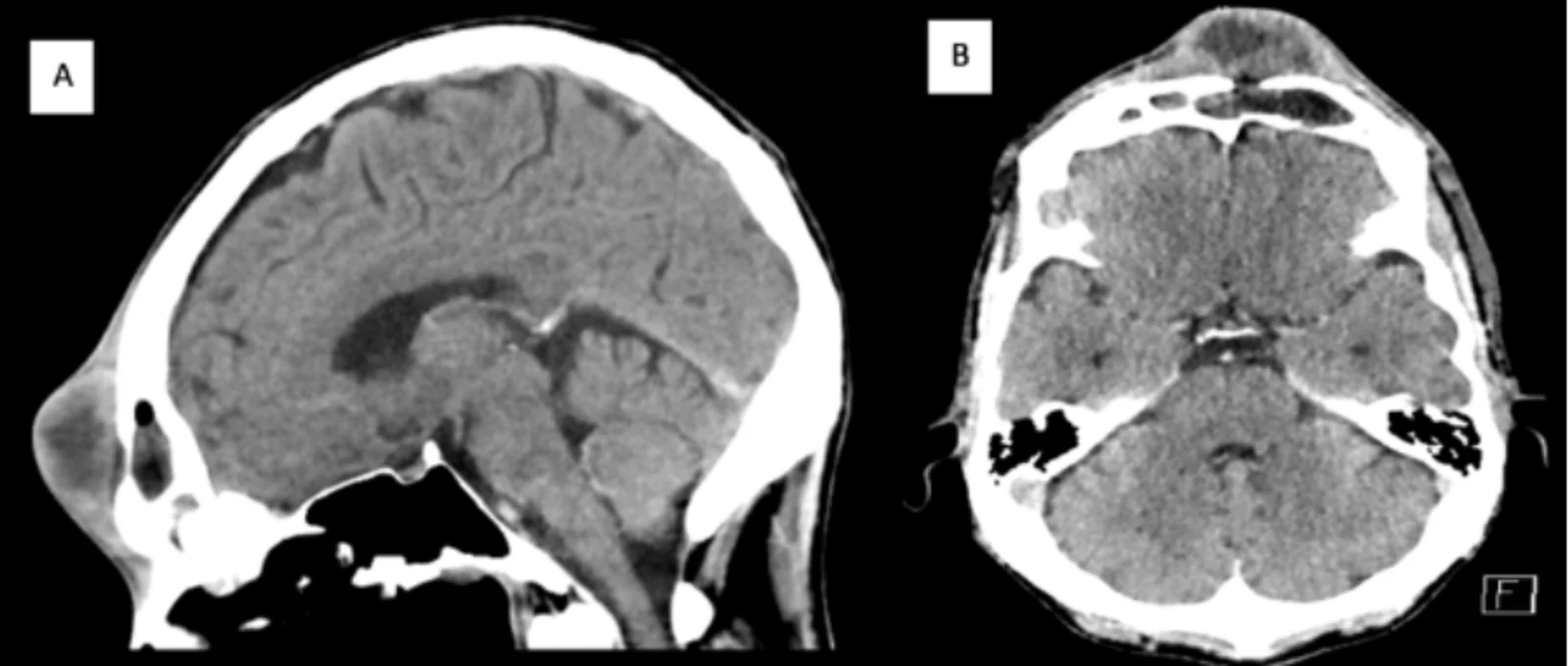
Sagittal (A) and axial (B) CT images demonstrating a frontal sinus mucocele with sinus expansion, anterior frontal and subperiosteal collections, and anterior and posterior table defects.

The diagnosis of PPT was made. Following discussion with a consultant microbiologist and in accordance with local guidelines, the patient was commenced on IV vancomycin and metronidazole, together with oral ciprofloxacin and IV dexamethasone. Definitive surgical drainage was subsequently undertaken at a tertiary ENT center, and the patient’s recovery was uncomplicated.

## Discussion

PPT was first described by Sir Percival Pott in 1768 in relation to head trauma and can be defined as osteomyelitis of the frontal bone with an associated subperiosteal abscess. Although initially associated with head trauma, it is now more commonly reported as a complication of frontal sinusitis. Dental infection commonly results in maxillary sinusitis and has been reported as a potential source of PPT. Consequently, a dental origin should be considered in patients presenting with unilateral maxillary sinusitis or a relevant dental history [3].

The condition has become rare in the antibiotic era, with most reported cases occurring in adolescents and young adults. More recent literature demonstrates that adult presentations, although uncommon, are well described. A systematic review of 125 adult cases reported a median age of 45 years, and a more recent systematic review and meta-analysis identified 181 adult cases with a mean age of 47 years [2,4].

Infection may spread by direct extension from the frontal sinus or through the diploic venous system, resulting in osteomyelitis and abscess formation. This process carries a significant risk of intracranial extension, including meningitis, epidural abscess, cerebral abscess, and venous sinus thrombosis [1]. Intracranial complications remain a significant concern in adults, with rates of 30.4% reported in one systematic review and 38.1% in a more recent meta-analysis [2,4].

This case demonstrates an important learning point for clinicians. The absence of systemic toxicity does not reliably exclude significant underlying osteomyelitis or abscess formation. Despite a large frontal collection, inflammatory markers were only moderately elevated, and the patient remained afebrile, with no neurological or visual abnormalities. Clinical stability should therefore not provide reassurance when the history and examination raise suspicion of deeper infective pathology. The differential diagnosis of frontal swelling in the ED is broad, and a superficial soft tissue infection may initially appear more likely. However, frontal swelling alongside persistent sinonasal symptoms or a history of dental infection should prompt consideration of PPT. This is particularly relevant in district general hospitals, where timely specialist transfer may need to be facilitated.

Contrast-enhanced CT is typically the initial investigation of choice and can demonstrate features including sinus disease, bony erosion, and associated collections. MRI provides better assessment of soft tissue and intracranial involvement and is particularly useful when intracranial complications are suspected.

The mainstay of treatment is urgent surgical drainage combined with prolonged antimicrobial therapy. Recent reviews continue to support an early, multidisciplinary approach involving otolaryngology, microbiology, and, where required, neurosurgical services [1].

## Conclusions

PPT is a rare but potentially serious complication of sinonasal and odontogenic infection that can occur in adults. Presentations may lack fever, systemic illness, neurological abnormalities, or markedly elevated inflammatory markers despite significant underlying disease. Emergency clinicians should consider PPT in patients presenting with frontal swelling, particularly when associated with preceding sinonasal or dental symptoms. Early recognition, urgent imaging, prompt antimicrobial therapy, and timely specialist referral are crucial to identifying and managing potentially serious intracranial complications.
